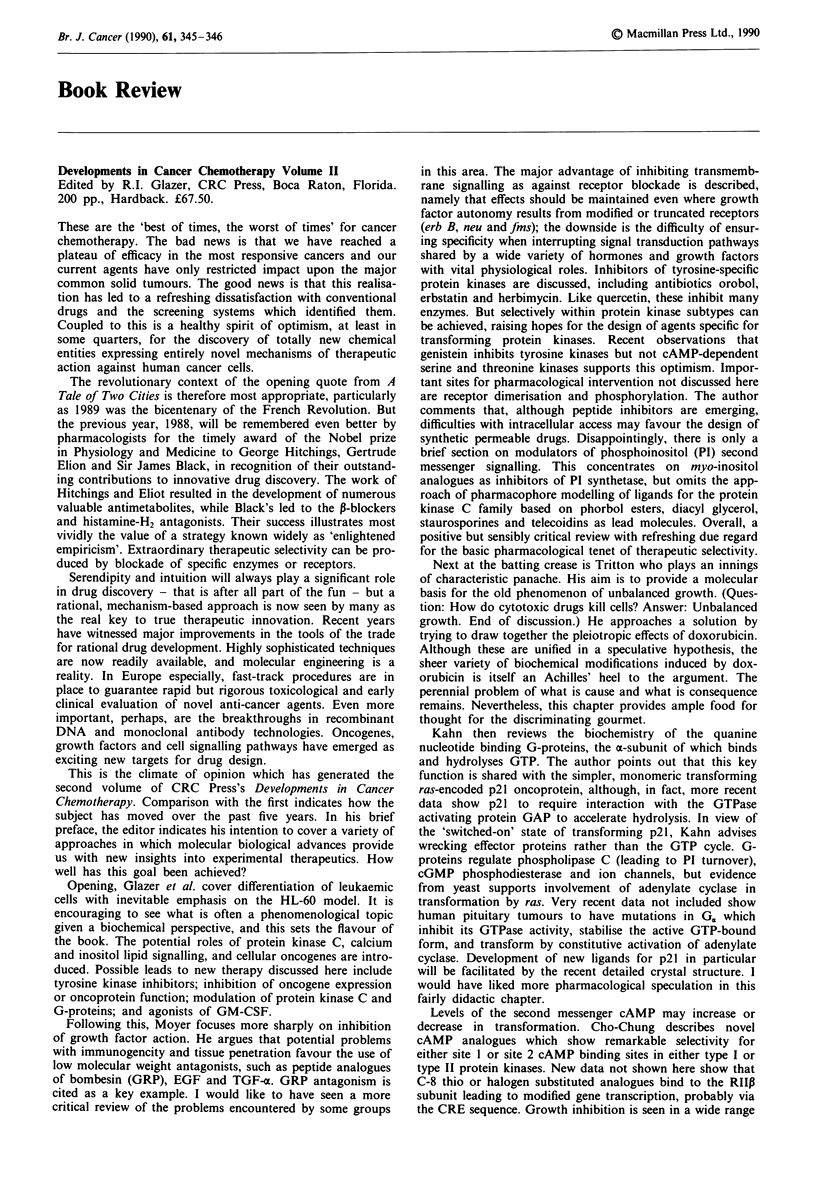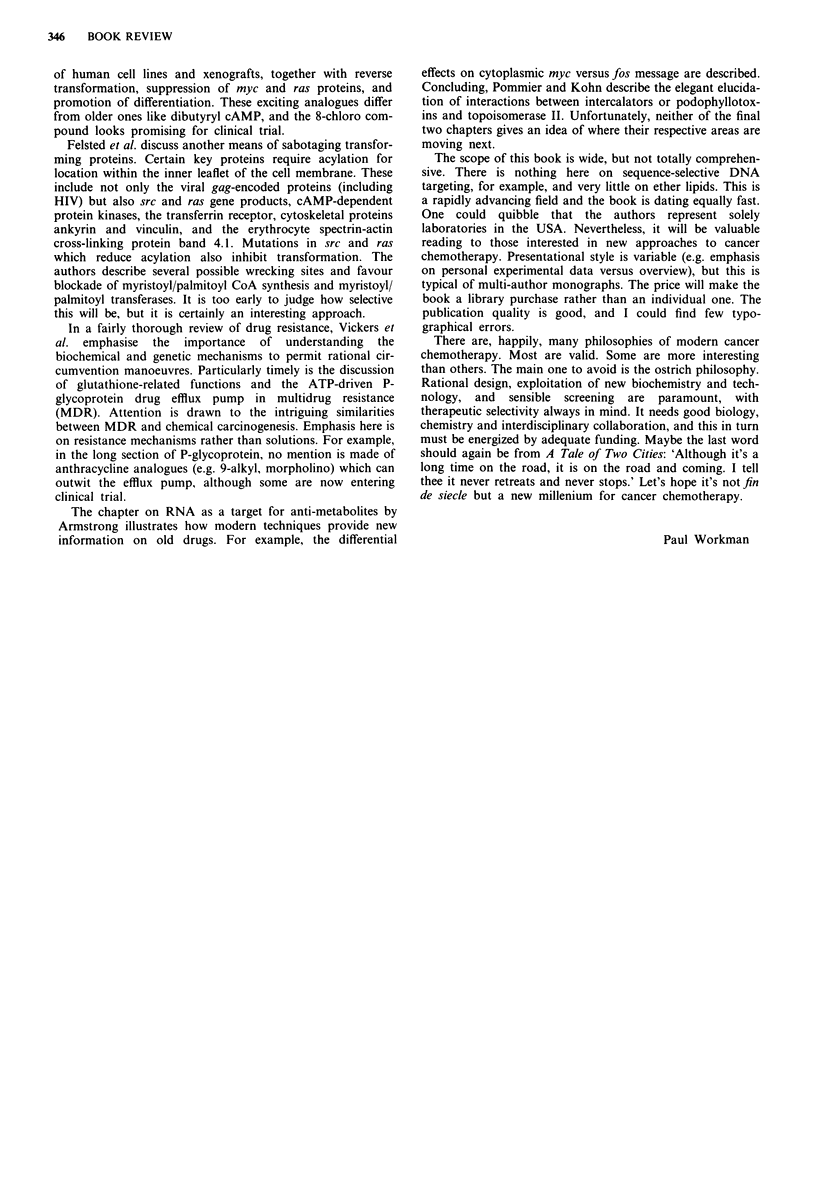# Developments in Cancer Chemotherapy Volume II

**Published:** 1990-02

**Authors:** Paul Workman


Br. J. Cancer (1990), 61, 345–346 © Macmillan Press Ltd., 1990

### Book Review

**Developments in Cancer Chemotherapy Volume II**
Edited by R.I. Glazer, CRC Press, Boca Raton, Florida. 200 pp., Hardback. £67.50.

These are the 'best of times, the worst of times' for cancer chemotherapy. The bad news is that we have reached a plateau of efficacy in the most responsive cancers and our current agents have only restricted impact upon the major common solid tumours. The good news is that this realisation has led to a refreshing dissatisfaction with conventional drugs and the screening systems which identified them. Coupled to this is a healthy spirit of optimism, at least in some quarters, for the discovery of totally new chemical entities expressing entirely novel mechanisms of therapeutic action against human cancer cells.

The revolutionary context of the opening quote from *A Tale of Two Cities* is therefore most appropriate, particularly as 1989 was the bicentenary of the French Revolution. But the previous year, 1988, will be remembered even better by pharmacologists for the timely award of the Nobel prize in Physiology and Medicine to George Hitchings, Gertrude Elion and Sir James Black, in recognition of their outstanding contributions to innovative drug discovery. The work of Hitchings and Eliot resulted in the development of numerous valuable antimetabolites, while Black's led to the β-blockers and histamine-$H_2$ antagonists. Their success illustrates most vividly the value of a strategy known widely as 'enlightened empiricism'. Extraordinary therapeutic selectivity can be produced by blockade of specific enzymes or receptors.

Serendipity and intuition will always play a significant role in drug discovery – that is after all part of the fun – but a rational, mechanism-based approach is now seen by many as the real key to true therapeutic innovation. Recent years have witnessed major improvements in the tools of the trade for rational drug development. Highly sophisticated techniques are now readily available, and molecular engineering is a reality. In Europe especially, fast-track procedures are in place to guarantee rapid but rigorous toxicological and early clinical evaluation of novel anti-cancer agents. Even more important, perhaps, are the breakthroughs in recombinant DNA and monoclonal antibody technologies. Oncogenes, growth factors and cell signalling pathways have emerged as exciting new targets for drug design.

This is the climate of opinion which has generated the second volume of CRC Press's *Developments in Cancer Chemotherapy*. Comparison with the first indicates how the subject has moved over the past five years. In his brief preface, the editor indicates his intention to cover a variety of approaches in which molecular biological advances provide us with new insights into experimental therapeutics. How well has this goal been achieved?

Opening, Glazer *et al.* cover differentiation of leukaemic cells with inevitable emphasis on the HL-60 model. It is encouraging to see what is often a phenomenological topic given a biochemical perspective, and this sets the flavour of the book. The potential roles of protein kinase C, calcium and inositol lipid signalling, and cellular oncogenes are introduced. Possible leads to new therapy discussed here include tyrosine kinase inhibitors; inhibition of oncogene expression or oncoprotein function; modulation of protein kinase C and G-proteins; and agonists of GM-CSF.

Following this, Moyer focuses more sharply on inhibition of growth factor action. He argues that potential problems with immunogencity and tissue penetration favour the use of low molecular weight antagonists, such as peptide analogues of bombesin (GRP), EGF and TGF-α. GRP antagonism is cited as a key example. I would like to have seen a more critical review of the problems encountered by some groups in this area. The major advantage of inhibiting transmembrane signalling as against receptor blockade is described, namely that effects should be maintained even where growth factor autonomy results from modified or truncated receptors (*erb B*, *neu* and *fms*); the downside is the difficulty of ensuring specificity when interrupting signal transduction pathways shared by a wide variety of hormones and growth factors with vital physiological roles. Inhibitors of tyrosine-specific protein kinases are discussed, including antibiotics orobol, erbstatin and herbimycin. Like quercetin, these inhibit many enzymes. But selectively within protein kinase subtypes can be achieved, raising hopes for the design of agents specific for transforming protein kinases. Recent observations that genistein inhibits tyrosine kinases but not cAMP-dependent serine and threonine kinases supports this optimism. Important sites for pharmacological intervention not discussed here are receptor dimerisation and phosphorylation. The author comments that, although peptide inhibitors are emerging, difficulties with intracellular access may favour the design of synthetic permeable drugs. Disappointingly, there is only a brief section on modulators of phosphoinositol (PI) second messenger signalling. This concentrates on *myo*-inositol analogues as inhibitors of PI synthetase, but omits the approach of pharmacophore modelling of ligands for the protein kinase C family based on phorbol esters, diacyl glycerol, staurosporines and telecoidins as lead molecules. Overall, a positive but sensibly critical review with refreshing due regard for the basic pharmacological tenet of therapeutic selectivity.

Next at the batting crease is Tritton who plays an innings of characteristic panache. His aim is to provide a molecular basis for the old phenomenon of unbalanced growth. (Question: How do cytotoxic drugs kill cells? Answer: Unbalanced growth. End of discussion.) He approaches a solution by trying to draw together the pleiotropic effects of doxorubicin. Although these are unified in a speculative hypothesis, the sheer variety of biochemical modifications induced by doxorubicin is itself an Achilles' heel to the argument. The perennial problem of what is cause and what is consequence remains. Nevertheless, this chapter provides ample food for thought for the discriminating gourmet.

Kahn then reviews the biochemistry of the quanine nucleotide binding G-proteins, the α-subunit of which binds and hydrolyses GTP. The author points out that this key function is shared with the simpler, monomeric transforming *ras*-encoded p21 oncoprotein, although, in fact, more recent data show p21 to require interaction with the GTPase activating protein GAP to accelerate hydrolysis. In view of the 'switched-on' state of transforming p21, Kahn advises wrecking effector proteins rather than the GTP cycle. G-proteins regulate phospholipase C (leading to PI turnover), cGMP phosphodiesterase and ion channels, but evidence from yeast supports involvement of adenylate cyclase in transformation by *ras*. Very recent data not included show human pituitary tumours to have mutations in $G_\alpha$ which inhibit its GTPase activity, stabilise the active GTP-bound form, and transform by constitutive activation of adenylate cyclase. Development of new ligands for p21 in particular will be facilitated by the recent detailed crystal structure. I would have liked more pharmacological speculation in this fairly didactic chapter.

Levels of the second messenger cAMP may increase or decrease in transformation. Cho-Chung describes novel cAMP analogues which show remarkable selectivity for either site 1 or site 2 cAMP binding sites in either type I or type II protein kinases. New data not shown here show that C-8 thio or halogen substituted analogues bind to the RIIβ subunit leading to modified gene transcription, probably via the CRE sequence. Growth inhibition is seen in a wide range of human cell lines and xenografts, together with reverse transformation, suppression of *myc* and *ras* proteins, and promotion of differentiation. These exciting analogues differ from older ones like dibutyryl cAMP, and the 8-chloro compound looks promising for clinical trial.

Felsted *et al.* discuss another means of sabotaging transforming proteins. Certain key proteins require acylation for location within the inner leaflet of the cell membrane. These include not only the viral *gag*-encoded proteins (including HIV) but also *src* and *ras* gene products, cAMP-dependent protein kinases, the transferrin receptor, cytoskeletal proteins ankyrin and vinculin, and the erythrocyte spectrin-actin cross-linking protein band 4.1. Mutations in *src* and *ras* which reduce acylation also inhibit transformation. The authors describe several possible wrecking sites and favour blockade of myristoyl/palmitoyl CoA synthesis and myristoyl/palmitoyl transferases. It is too early to judge how selective this will be, but it is certainly an interesting approach.

In a fairly thorough review of drug resistance, Vickers *et al.* emphasise the importance of understanding the biochemical and genetic mechanisms to permit rational circumvention manoeuvres. Particularly timely is the discussion of glutathione-related functions and the ATP-driven P-glycoprotein drug efflux pump in multidrug resistance (MDR). Attention is drawn to the intriguing similarities between MDR and chemical carcinogenesis. Emphasis here is on resistance mechanisms rather than solutions. For example, in the long section of P-glycoprotein, no mention is made of anthracycline analogues (e.g. 9-alkyl, morpholino) which can outwit the efflux pump, although some are now entering clinical trial.

The chapter on RNA as a target for anti-metabolites by Armstrong illustrates how modern techniques provide new information on old drugs. For example, the differential effects on cytoplasmic *myc* versus *fos* message are described. Concluding, Pommier and Kohn describe the elegant elucidation of interactions between intercalators or podophyllotoxins and topoisomerase II. Unfortunately, neither of the final two chapters gives an idea of where their respective areas are moving next.

The scope of this book is wide, but not totally comprehensive. There is nothing here on sequence-selective DNA targeting, for example, and very little on ether lipids. This is a rapidly advancing field and the book is dating equally fast. One could quibble that the authors represent solely laboratories in the USA. Nevertheless, it will be valuable reading to those interested in new approaches to cancer chemotherapy. Presentational style is variable (e.g. emphasis on personal experimental data versus overview), but this is typical of multi-author monographs. The price will make the book a library purchase rather than an individual one. The publication quality is good, and I could find few typographical errors.

There are, happily, many philosophies of modern cancer chemotherapy. Most are valid. Some are more interesting than others. The main one to avoid is the ostrich philosophy. Rational design, exploitation of new biochemistry and technology, and sensible screening are paramount, with therapeutic selectivity always in mind. It needs good biology, chemistry and interdisciplinary collaboration, and this in turn must be energized by adequate funding. Maybe the last word should again be from *A Tale of Two Cities*: 'Although it's a long time on the road, it is on the road and coming. I tell thee it never retreats and never stops.' Let's hope it's not *fin de siecle* but a new millenium for cancer chemotherapy.

Paul Workman